# Evaluation of a multicomponent pathway to address inpatient delirium on a neurosciences ward

**DOI:** 10.1186/s12913-018-2906-3

**Published:** 2018-02-12

**Authors:** Ethan G. Brown, S. Andrew Josephson, Noriko Anderson, Mary Reid, Melissa Lee, Vanja C. Douglas

**Affiliations:** 1 Department of Neurology, University of California, San Francisco, CA USA; 2 Department of Neurology, University of California, Irvine, USA

**Keywords:** Delirium, Patient safety, Outcome research, Cognitive aging

## Abstract

**Background:**

Delirium is a frequent and detrimental complication of inpatient hospitalization. Multicomponent intervention in selected groups has been shown to prevent and treat delirium, though little data exists on the effect of intervention in neurological patients. We studied the efficacy of a multicomponent delirium care pathway implemented on a largely neurology and neurosurgery hospital ward among unselected patients.

**Methods:**

We incorporated a multicomponent delirium care pathway into the workflow of a university hospital for patients older than 50 years. The pathway involved risk-stratification for development of delirium, delirium screening, and non-pharmacologic behavioral prevention and intervention. We then retrospectively reviewed admissions before and after implementation of the care pathway. Our primary endpoint was incidence of delirium; secondary endpoints included delirium days, length of stay, restraint use, readmission rates, and discharge disposition.

**Results:**

Seven hundred ninety eight admissions from before the delirium care pathway went into effect and 797 admissions from afterwards were reviewed. Baseline characteristics between groups were similar. Delirium incidence between the two groups did not change (7.0% before vs 7.2% after, *p* = 0.89). Length of stay among delirious patients significantly decreased after implementation of the delirium care pathway (9.60 before vs 7.06 after, β = − 0.16, adjusted *p*-value = 0.001).

**Conclusion:**

Implementation of a delirium care pathway on a neurosciences ward was not associated with changes in the rate of delirium development, though length of stay among delirious patients decreased. In a largely neurologic population, multicomponent intervention to prevent and treat delirium may not change delirium incidence, but may be effective in mitigating delirium complications.

## Background

Multi-component, non-pharmacologic interventions both prevent the development and mitigate the complications of inpatient delirium [1, 2], but their efficacy has not been studied in neurologic populations. Neurologic patients are at high risk of delirium, with neurologic emergency rooms, wards, and stroke units seeing rates of delirium ranging from 11 to 16% [3–5]. Delirium has been associated with a longer length of stay on a neurology ward, worse outcome after stroke, and acceleration of cognitive decline in patients with dementia [3, 5, 6].

Over the last decade, multi-component interventions implemented by hospital staff have been shown to reduce rates and detrimental outcomes of delirium in many settings [2, 7]. These interventions include frequent reorientation, early mobilization, improvement in sleep regulation, ensuring adequate hydration, treating urinary retention and constipation, and ensuring hearing and visual aids are present if needed [1].

Despite the efficacy of the multicomponent approach, few studies have examined these interventions in patients with neurologic pathology. Delirium in neurology and neurosurgery patients may be similar to general medicine cases. On the other hand, acute confusion in the setting of an underlying lesion in the central nervous system may be much less modifiable with non-pharmacologic interventions. Furthermore, the systematic changes that previous protocols have implemented require increased staffing to help with screening and treatment. A protocol that does not require any additional staff would be ideal, and may be most feasible on a neurosciences unit where nurses and other staff are specifically trained in cognitive assessment.

To address these issues, we developed a delirium care pathway for the neurosciences ward at a university medical center. The care pathway screened for the risk and presence of delirium, and implemented non-pharmacologic interventions to those patients at high risk of developing delirium and to those who had already developed delirium. To measure the efficacy of this intervention, we compared the incidence of delirium and its secondary clinical outcomes before and after the pathway was put in place.

## Methods

We conducted a retrospective cohort study investigating the clinical efficacy of a comprehensive delirium care pathway instituted on the neurosciences ward of the University of California San Francisco Medical Center. The intervention was implemented on the neurosciences ward as part of a department-wide effort to improve delirium care. The study was planned prior to implementation of the care pathway, but data collection and analysis was retrospective. The ward is staffed with nurses trained in neurological assessment, and patients are largely neurology and neurosurgery, though patients on other services may board on this floor as well.

### Intervention

**Fig. 1:**
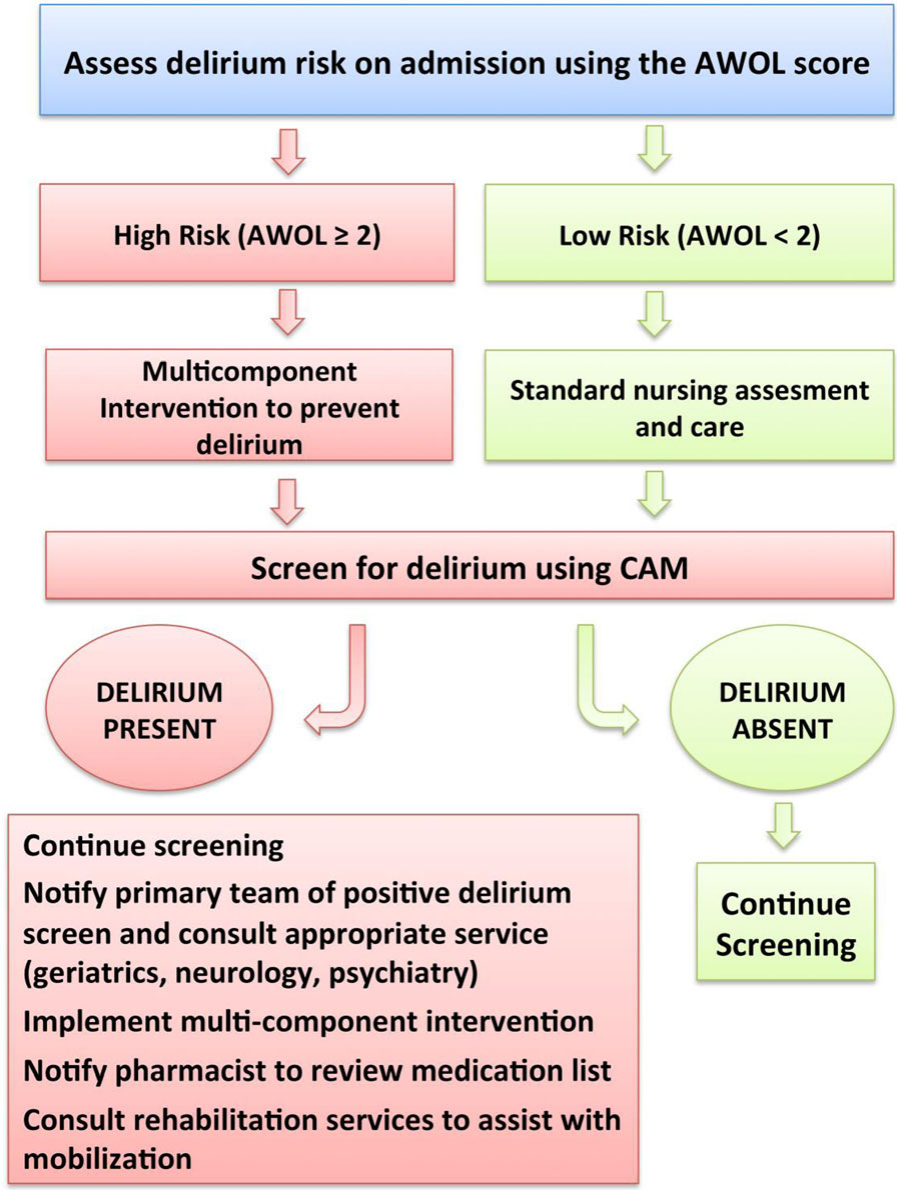
The delirium care pathway (DCP) flow diagram. See text for description of AWOL. CAM = confusion assessment method

All patients who were admitted to the neurosciences floor and were over the age of 50 years were enrolled in the care pathway, as depicted in Fig. 1. First, nursing staff would calculate the AWOL prediction score, which stratifies patients based on their risk of delirium [8]. While this score was historically developed and initially validated in hospitalized medical patients, we separately validated the usefulness of this score in neurological patients using a subset of patients in this cohort [9]. In our study the area under the receiver operating characteristic curve for predicting delirium was 0.83, suggesting good predictive value. If a patient was found to be at increased risk of developing delirium (defined as an AWOL score ≥ 2, based on maximizing sensitivity and specificity from previous studies [8, 9]), staff would employ evidence-based non-pharmacologic delirium prevention measures, including but not limited to frequent re-orientation, regulating sleep-wake cycles, and reducing restraints, lines, and catheters as possible. These strategies were derived from the National Institute for Health and Care Excellence (NICE) Guidelines [10].

While application of these guidelines to all patients would be ideal, this algorithm served to focus nursing efforts on particularly high-risk patients. All patients, regardless of risk-level, were screened for delirium by their nurse once during every twelve-hour shift using the confusion assessment method (CAM), a validated method for detecting delirium [11]. If a patient screened positive for delirium, his or her nurse would notify the primary team, the pharmacist, and initiate a neurology consultation to assist with management of delirium. In addition, they would ensure non-pharmacologic management strategies were taking place. Pharmacy and neurology staff had discussions to coordinate recommendations regarding delirium care, with attention to avoiding high-risk medications and addressing common exacerbating factors.

### Study design

The delirium care pathway was started on November 1st, 2013. To investigate its effect, we examined patients admitted before and after the care pathway was implemented. Our ‘before’ study period included admissions from November 1st, 2012 through October 31st, 2013; our ‘after’ group began following a run-in period of 6 months, spanning April 1st 2014 through March 30th, 2015. Any patient whose date of admission to the neurosciences ward fell during these epochs and was over 50 years old was included as a potential subject.

### Case ascertainment

After implementation of the care pathway, nursing staff identified cases of delirium through use of the CAM, but for this study delirium was identified without consideration of the CAM. Rather, delirium was identified based on any reported mental status changes documented in the notes of the chart. We chose this method for two reasons: first, before the delirium care pathway intervention, none of the subjects had CAM scores, and we wanted a consistent method for detecting delirium between the two groups. Secondly, active screening through the CAM could naturally increase the detection rate of delirium, leading to over diagnosis in the cohort after the pathway was introduced. Health care provider documentation, while potentially influenced by the care pathway, should be less affected, and still communicate similar information about the patient in both settings.

This method of chart review to detect delirium has been previously validated with a sensitivity of 74% and specificity of 83% when compared to interviewer-based methods [12]. Chart reviews were performed by one investigator (E.G.B.). A subset of charts was reviewed by the senior investigator (V.C.D.) to internally validate the methodology. Charts where the delirium diagnosis was uncertain were adjudicated by the investigators. Blinding to the care epoch was not possible because the medical record could not be reviewed with the dates removed. All physician and nursing notes were reviewed for the presence of absence of delirium, the start and end dates, the subtype (hyperactive versus hypoactive), who diagnosed the delirium, and any potential cause if identified. To assess the accuracy of the chart review method, delirium diagnosis by chart review was compared to the CAM assessment of bedside nurses. We also reviewed the past medical history available in the notes and problem list to calculate a Charlson comorbidity index [13], and made special note of a history of psychiatric disease or epilepsy. If the family or admitting physician noted a history of long-term difficulty with thinking, or if there was a formal diagnosis of dementia in the problem list, then the subject was recorded as having prior cognitive impairment. While delirium may be a particularly detrimental complication in patients with dementia and identifying this subgroup is particularly important [6], the level of detail available in the chart did not allow for the distinction from those patients with mild cognitive impairment. We also collected demographic information, dates of admission, transfers, and discharge, hospital service at admission, 30-day readmission rates to our medical center, presence and duration of restraint or sitter use, and discharge disposition. The results of the AWOL prediction score and the CAM were extracted. In delirious patients, the use of antipsychotics, benzodiazepines, or other sleep aids was recorded.

### Delirium prevalence vs incidence

Our primary outcome was the change in incidence of hospital-acquired delirium on the neurosciences unit before and after the delirium care pathway was implemented. As the delirium care pathway was in use only on the neurosciences ward, we restricted the primary outcome to delirium that developed on that floor only, or ‘Incident Delirium.’ A subject was classified with incident delirium if he or she was admitted to the neurosciences ward without any mental status abnormality, and subsequently developed delirium while on the neurosciences ward. Any subject who was admitted.

or transferred to the neurosciences unit having already developed delirium, either before admission or on another unit, was considered ‘prevalent delirium.’ While the primary outcome was limited to incident delirium, secondary outcomes, which included delirium duration, overall length of stay, restraint use, sitter use, disposition to nursing facility, and 30-day readmission rate, were analyzed in subjects with either incident or prevalent delirium, as we expected the care pathway to have some beneficial effect on all cases of delirium. Restraint and sitter use included use of these measures on the neurosciences ward only.

### Statistical analysis

Previous studies have shown that non-pharmacologic interventions can prevent one third of delirium cases. Therefore our sample size calculation was based on an estimated reduction of delirium incidence from 12% to 8% of inpatient hospitalizations, with α = 0.05 and β = 0.80; to show this difference required review of 1600 hospital admissions, 800 from before the delirium care pathway and 800 from afterwards. We did not restrict the admissions to unique patients, as we wanted to assess the care pathway in all patients, regardless of any history of delirium or hospital admission. Furthermore, we could not record recent admissions or delirium episodes in other hospitals, so restricting the population to unique patients may have biased our sample. Overall, there were 2138 eligible admissions before the care pathway and 2151 potential admissions afterwards. We randomly selected 800 admissions from before (using the “rand” function in Microsoft Excel 2010 version 2.0). We then sorted the admissions from after the care pathway into 10-year age groups (50–59, 60–69, 70–79, 80–89, and > 90). Finally, we randomly selected the same number of subjects from within each age group as were represented in the admissions from before the pathway, in order to have similar age distributions in each cohort.

Because some of our analyses involved some re-admissions of the same patient, we could not assume each observation was independent and needed to account for repeated measures in evaluation of our outcome. For analyses that included repeated measures we used a generalized estimating equations model (GEE), which accounts for potential correlations among clustered observations. A logistic link function was used for binary outcomes. For continuous outcomes such as length of stay, an identity link function was used. We used natural log transformations of continuous variables to reduce positive skew and to approximate a normal distribution, generating ratios of geometric means. For all outcomes where repeated admissions were excluded, such as our descriptive data in Tables 1 and 2, a Chi-square test or Wilcoxon’s rank sum test was used for categorical or non-parametric continuous data, respectively. A bivariate analysis was done to search for confounders. In addition to any potential confounders, we performed multivariable models with clinically relevant factors including age at admission, gender, the presence of prior cognitive impairment, and Charlson comorbidity index [13]. Many of these characteristics have been previously identified as risk factors for delirium and were therefore kept in our model [14]. For length-of-stay, we ran secondary multivariable regressions using the outcome variable without outliers (defined as more than 3 standard-deviations from the mean) and after log-transformation, both with similar significant results. Given that we were testing 9 hypotheses, we used the Bonferroni correction and statistical significance for all outcomes was set at α < (0.05/9) = 0.006. All statistical analyses were performed using a licensed copy of STATA, version 13.1 [15].

**Table 1:** Subject characteristics before and after implementation of the delirium care pathway in all patients

	Before Care Pathway (*n* = 752 patients)	After Care Pathway (*n* = 749 patients)	*p*-value
Age, median (IQR)	67 (59–74)	67 (58–75)	0.89
Female, no. (%)	399 (53)	380 (51)	0.37
Race, no. (%)			0.365
White	470 (63)	456 (61)	
Black	49 (6.5)	49 (6.5)	
Asian / Pacific Islander	129 (17)	130 (17)	
Other / Unknown	104 (14)	114 (15)	
Hispanic	70 (9.3)	61 (8.1)	0.027
Modified Charlson comorbidity score, median (IQR)	4 (2–5)	4 (2–5)	0.22
Prior cognitive impairment, no. (%)	119 (16)	115 (15)	0.80
Primary Hospital Service, no. (%)			0.64
Neurology	163 (22)	168 (22)	
Neurosurgery	359 (48)	365 (49)	
Hospital medicine	137 (18)	138 (18)	
Other	93 (12)	78 (10)	

*mCCM* modified Charlson comorbidity score

**Table 2:** Subject characteristics before and after implementation of the delirium care pathway in delirious patients only

	Before Care Pathway (*n* = 197 patients)	After Care Pathway (*n* = 194 patients)	*p*-value
Age, median (IQR)	71 (62–80)	72 (64–81)	0.77
Female, no. (%)	112 (57)	96 (49)	0.14
Race, no. (%)			0.92
White	111 (56)	107 (55)	
Black	12 (6.1)	11 (5.7)	
Asian / Pacific Islander	42 (21)	50 (26)	
Other / Unknown	32 (16)	26 (13)	
Hispanic	23 (12)	13 (6.7)	0.24
Modified Charlson comorbidity score, median (IQR)	5 (3–6)	5 (3–6)	0.83
Prior cognitive impairment, no. (%)	65 (33)	61 (31)	0.74
Primary Hospital Service, no. (%)			0.014
Neurology	54 (27)	65 (34)	
Neurosurgery	102 (52)	91 (47)	
Hospital medicine	29 (15)	36 (19)	
Other	12 (6.1)	2 (1.0)	

*mCCM* modified Charlson comorbidity score

## Results

### Description of subjects

Of 1600 charts chosen for review, 798 admissions were reviewed in the pre-intervention group (two charts were restricted), and 797 in the after-group (two charts were restricted, and one subject was under 50 years old at time of admission). The mean age of the groups was similar (67.1 +/− 11.1 before and 67.1 +/− 11.2 after, *p* = 0.89), as were the ratio of men to women and the rate of cognitive impairment prior to admission, though there were more Hispanic patients in the pre-intervention group (Table 1). More than 2/3 of patients were on either the neurosurgical or neurology service in both epochs. Characteristics in patients with delirium only are shown in Table 2. There was a significant difference between hospital services in the pre-intervention group compared to the post-intervention group (*p* = 0.014) among delirious patients only, but otherwise demographic information was similar between epochs.

### Incidence and prevalence of delirium

Overall, 213 (27%) patient admissions involved delirium before implementation of the delirium care pathway, while 204 (26%) of patient admissions involved delirium afterwards (Table 3). Slightly more involved prevalent delirium in the pre-intervention group, though the difference was not significant (21% vs 20%, *p* = 0.60). The overall rate of incident delirium did not change between admissions before and after the delirium care pathway was implemented (7.0% before the delirium care pathway vs 7.2% after, *p* = 0.53).

**Table 3:** Outcomes before and after implementation of the delirium care pathway among all admissions

	Before care pathway (*n* = 798 admissions)	After care pathway (*n* = 797 admissions)	*p*-value
Prevalent Delirium, no. (%)	169 (21)	158 (20)	0.60
Incident Delirium, no. (%)	44 (7.0)	46 (7.2)	0.53
LOS, mean days (SD)	5.2 (6.2)	4.5 (4.1)	0.339
Restrained, no. (%)	38 (4.8)	30 (3.8)	0.52
Days restrained, mean days (SD)	5.7 (6.2)	4.3 (4.5)	0.305
Sitter, no. (%)	89 (11)	81 (10)	0.31
Readmissions, no. (%)	69 (8)	64 (8)	0.53
Disposition to SNF, no. (%)	88 (11)	95 (12)	0.36

*LOS* length of stay, *SNF* skilled nursing facility

While the majority of the patients in whom the care pathway was implemented were neurology and neurosurgery patients, a significant proportion (28%) were non-neurology patients. To examine the effect of the care pathway in neurology and neurosurgery patients alone, we repeated the analysis in admissions to neurology and neurosurgical services (*n* = 552 before implementation and *n* = 571 afterwards). Neither prevalence nor incidence of delirium significantly changed (prevalence: 25% before and 21% after, *p* = 0.24; incidence: 7.7% before and 8.9% after, *p* = 0.54). We also evaluated the care pathway among cognitively impaired patients, a particularly high-risk group (*n* = 126 admissions before and after implementation of the care pathway). The results were similar (prevalence: 44% before and 43% after, *p* = 0.72; incidence: 18.6% before and 18.1% after, *p* = 0.97).

### Secondary outcomes

For secondary outcomes, we examined subjects with delirium only. Length of stay decreased by more than 2 days among delirious patients after the delirium care pathway was implemented, even after adjustment for age, gender, prior cognitive impairment, and comorbidity (9.60 vs 7.06, β = − 0.072, adjusted *p*-value = 0.008; Table 4). The relationship was still significant (β = − 0.076, adjusted *p*-value = 0.005) after further adjustment for inpatient hospital service.

**Table 4:** Outcomes before and after implementation of the delirium care pathway in admissions involving delirium only

	Before care pathway (*n* = 213 admissions)	After care pathway (*n* = 204 admissions)	Unadjusted *p*-value
LOS, mean days (SD)	9.60 (9.3)	7.05 (5.7)	0.013
Restrained, no. (%)	36 (17)	29 (14)	0.57
Days restrained on the ward, mean days (SD)	5.8 (6.4)	4.4 (4.5)	0.337
Sitter, no. (%)	67 (31)	51 (25)	0.18
Readmissions, no. (%)	24 (11)	11 (5.4)	0.16
Disposition to SNF, no. (%)	55 (26)	55 (27)	0.76
Delirium Days on ward, mean days (SD)	5.4 (5.7)	4.2 (3.5)	0.947

Importantly, length of stay in patients without delirium did not significantly change (3.6 +/− 3.4 before and 3.7 +/− 2.9, *p* = 0.75). There was a reduction in delirium days after the care plan was implemented, though this difference was not statistically significant after adjusting for age, comorbidity, gender, and cognitive impairment (5.4 vs. 4.2 days, *p* = 0.647; Table 4). We also examined these outcomes in delirious neurology and neurosurgery patients alone. In this population length of stay similarly shortened, from 11.0 days to 7.7 days (β = − 0.10, adjusted *p*-value < 0.001). Length of stay in non-delirious patients in this population did not change (4.0 vs 3.8 days, *p* = 0.339). Among delirious patients with prior cognitive impairment, length of stay decreased from 8.2 days to 6 days, though this change was not significant (adjusted p-value = 0.25). Still, this represented a larger change than in non-delirious patients with prior cognitive impairment (4.5 vs 4.0, *p* = 0.45). Finally, as not all of the admissions involved unique cases, we performed a sensitivity analysis including only the first admission for delirious patients (*n* = 389 patients). We found that length of stay similarly decreased when only these hospital stays were included (9.50 days before vs 7.06 days after, β = − 0.17, adjusted *p*-value = 0.001).

Restraint use (17% before vs 14% after, *p* = 0.57) and sitter use (31% before vs 25% after, *p* = 0.18) both showed non-significant declines after implementation of the care pathway (Table 4). Thirty-day readmission rates decreased from 11% before the pathway to 5.4% afterwards, though the difference was not statistically significant (*p* = 0.16).

### Assessment of case ascertainment

Finally, we assessed our method of identifying cases of delirium through chart review, as this method has only been previously validated in general medical patients [12]. Compared to the diagnosis of delirium by the CAM performed by bedside nurses our chart-review delirium method showed a sensitivity of 76.3% and a specificity of 80.5%.

## Discussion

We demonstrated successful implementation of a multicomponent delirium care pathway on a neurosciences ward. While we did not find a change in the incidence of delirium after initiation of the care pathway, a reduced length of stay was observed among delirious patients, significant at our Bonferroni corrected threshold of an adjusted *p* < 0.006. These findings held true when the analysis was restricted to only neurology and neurosurgery patients.

The lack of reduction in incidence is unusual for a multicomponent pathway [7], and could be due to a number of reasons. One explanation may lie in the unique patient population of our study. The patients were primarily neurology and neurosurgery patients, with some hospital medicine and a minority of other surgical patients. As opposed to a general medical or surgical ward, where multi-component delirium interventions have been studied, delirium on a neurosciences ward may be less affected by addressing traditional risk factors. For instance, confusion from infection on a medical ward or immobility on an orthopedic surgery ward may be prevented by rehydration or physical therapy, respectively. On the other hand, altered mental status from an intracranial mass or post-ictal encephalopathy may occur regardless of all non-pharmacologic prevention efforts. This difference is important to consider for future multicomponent intervention models.

The incidence may also have been affected by the study design: as the care pathway included routine screening for delirium, increased awareness and detection of delirium may have led to an increase in identification of delirium in the post-intervention group, despite our attempt to mitigate detection bias by using a chart review method to identify delirium in both the pre- and post-intervention groups. However, because the incidence was similar between the two groups, either the care pathway had no effect on incidence or a beneficial effect was masked by an increase in delirium identification after the intervention.

Even if delirium incidence is not modifiable in neuroscience patients, multi-component intervention may mitigate the severity of delirium and its secondary effects regardless of its cause. In our study, the reduction in length of stay after the introduction of the care pathway is encouraging. It is unlikely that length of stay was reduced simply because of a general effort to discharge patients earlier over the time period of the study, since there was no reduction in length of stay for non-delirious patients. Differences in the study population likely did not contribute, as adjusted models were also significant. Finally the lack of increase in readmission rates indicates that patients were not necessarily discharged early inappropriately. A lack of increase in length of stay among delirious neurological patients and patients with prior cognitive impairment is reassuring and suggests that the care pathway may be effective in these settings.

The reduction of length of stay of more than 2 days likely diminishes risk of further inpatient complications and caregiver stress while substantially reducing costs. Reduced length of stay is associated with lower likelihood of readmission and lower all-cause mortality over time in medical patients [16]. In delirium, previous multicomponent interventions that reduced length of stay to a similar degree saw significant cost savings, even when the pathway involved additional staffing [17]. Cost of hospitalization was not available for our study, however the national average cost of a day in the hospital for patients over 45 years old is $2480, according to 2012 estimates [18]. With 2151 hospitalizations on our unit over the year we examined, and using the rate of delirium we detected of 26%, a 2.5-day reduction in hospital stay translates to savings of 3.4 million dollars yearly. While an estimate, the true savings are likely substantial, especially in the face of no added cost without any additional staff.

The reduction in length of stay may reflect a more rapid resolution of delirium with the care pathway, as suggested by the non-significant reduction in delirium duration we found after the care pathway was implemented. Our care pathway placed a particular emphasis on reducing the use of sedatives to treat delirium, which may have been a factor in reducing delirium duration, contributed to improved return to normal sleep-wake cycles, and increased opportunities for early mobility. The reduction in length of stay may also have been related to an increased level of comfort with discharging patients who were still mildly delirious, due to education of caregivers, nurses, and providers that delirious patients generally fare better at home than in hospital once the underlying cause of their delirium has been addressed.

The care pathway was associated with improvement in most secondary outcomes, although only the reduction in readmission rate showed a *p* < 0.05 in univariate analysis, and no secondary outcomes were significant at our more stringent statistical cutoff. Still, the trend for many of these is positive, including the number of delirium days, the use of sitters and the use of restraints. These are all clinically meaningful differences, and the study was underpowered to show changes in such infrequent events. Other studies evaluating multicomponent interventions for delirium have often focused on different secondary outcomes, and not highlighted these features of the hospitalization [7]. Bakker et al. [19] reported a non-significant increase in readmission after non-pharmacologic intervention. Chong et al. [20] reduced restraints completely, though only through the use of separate geriatrics unit; Avendano-Cespedes et al. [21] reported a non-significant trend towards less restraint use in their multidisciplinary intervention. Overall the findings in our study are encouraging, and they reinforce the need for these characteristics to be an outcome in future studies.

While several previous studies have shown improvement in delirium after incorporation of a multicomponent pathway [1, 22, 23], the delirium care pathway we implemented has some advantages. We did not require additional staffing for the behavioral interventions and screening, or the use of a specialized geriatric ward. Both medical and surgical patients were included, and nursing staff used a risk stratification tool that required very little additional patient assessment (< 2 min [8]), which allowed for efficient triaging of resources. These elements make the delirium care pathway more efficient for nursing staff with limited resources and time, allowing for more efficient integration of the protocol into hospital workflow.

Our study had several important limitations. We used a chart review method for detecting delirium, which was initially validated in an elderly medical population and therefore may not be generalizable to our population [12]. As we describe above, the sensitivity and specificity in our cohort, when using the CAM as the gold standard, were similar to that found the original study. In further support of chart abstraction as a mechanism for identifying delirium, a similar method was validated in a separate population, using the medical records system of the United Kingdom [24]; in this study, the sensitivity and specificity of identifying probable delirium was 58 and 93%, respectively. While the chart-review method may mischaracterize patients from lack of documentation, later studies have shown some benefits of using chart review, such as being able to evaluate the patient at multiple times of day, a challenging breadth for interview-based methods to achieve [25].

Still the chart-review method has reduced sensitivity, which may have limited our ability to detect all delirium cases. However, this likely affected the measured delirium incidence in both epochs equally, unless increased nursing screening led to more recognition and documentation of delirium in the chart notes. Detection of more subtle cases after care pathway implementation may have led to a falsely low change in delirium incidence but falsely high change in length of stay. On the other hand, an increased rate of false-negatives in the period before care pathway implementation may have led us to underestimate our length of stay for these patients, making the true effect of the pathway larger than reported. We tried to make our detection rates as similar as possible between epochs by having one investigator review all charts (E.G.B.), defining delirium as objectively as possible (based on key words in the chart), not using the CAM to detect delirium, and adjudicating challenging cases. Ideally the investigator reviewing the charts would be blinded to group, but the nature of chart review precluded this strategy.

Furthermore, compliance with individual components of the care pathway was not measured during the study. Therefore we were unable to test whether patients who received more aspects of the multi-component intervention were more likely to have improved outcomes. Finally, as with any study using a before/after design, we cannot rule out the observation of reduced length of stay being due to other unmeasured variables. These limitations would be better controlled through a randomized controlled trial, which would be helpful in this population of patients to understand the benefit of multicomponent intervention.

The need for addressing delirium during inpatient hospitalization is clear, and based on available evidence multicomponent pathways are currently our safest and most effective tool. This study suggests implementation of such a pathway on a neurosciences ward can modify the outcomes of delirium, particularly length of stay, but also calls attention to the potential differences between neurological patients and general medical or surgical patients.

## Conclusions

A multicomponent delirium care pathway involving active risk stratification and screening of delirium was introduced on the neurosciences ward at our institution. We did not find any changes in delirium incidence after the care pathway was implemented, but length of stay significantly decreased among delirious patients, while other secondary outcomes such as readmissions and sitter utilization showed a trend towards improvement. This suggests that multicomponent interventions to prevent and treat delirium in a population of largely neurology inpatients may not change delirium incidence, but may be effective in mitigating its complications.

## Abbreviations

AWOL: Delirium prediction tool. Calculated as Age > 80, inability to spell World backwards, disOrientation, and iLlness severity. See [8] for more details
CAM: Confusion assessment method
DCP: Delirium care pathway
OR: Odds ratio
